# The Complex Roles and Therapeutic Implications of m^6^A Modifications in Breast Cancer

**DOI:** 10.3389/fcell.2020.615071

**Published:** 2021-01-11

**Authors:** Min Wei, Jing-Wen Bai, Lei Niu, Yong-Qu Zhang, Hong-Yu Chen, Guo-Jun Zhang

**Affiliations:** ^1^Department of Breast and Thyroid Surgery, Xiang’an Hospital of Xiamen University, School of Medicine, Xiamen University, Xiamen, China; ^2^Cancer Research Center, School of Medicine, Xiamen University, Xiamen, China; ^3^Department of Oncology, Xiang’an Hospital of Xiamen University, School of Medicine, Xiamen University, Xiamen, China

**Keywords:** N^6^-methyladenosine, breast cancer, m^6^A modification regulator, mechanism pathways, FTO inhibitor

## Abstract

Accumulating evidence indicates that N^6^-methyladenosine (m^6^A), which directly regulates mRNA, is closely related to multiple biological processes and the progression of different malignancies, including breast cancer (BC). Studies of the aberrant expression of m^6^A mediators in BC revealed that they were associated with different BC subtypes and functions, such as proliferation, apoptosis, stemness, the cell cycle, migration, and metastasis, through several factors and signaling pathways, such as Bcl-2 and the PI3K/Akt pathway, among others. Several regulators that target m^6^A have been shown to have anticancer effects. Fat mass and obesity-associated protein (FTO) was identified as the first m^6^A demethylase, and a series of inhibitors that target FTO were reported to have potential for the treatment of BC by inhibiting cell proliferation and promoting apoptosis. However, the exact mechanism by which m^6^A modifications are regulated by FTO inhibitors remains unknown. m^6^A modifications in BC have only been preliminarily studied, and their mechanisms require further investigation.

## Introduction

Understanding the origins of cancer has changed significantly in recent decades, from being considered solely a genetic disease to being considered a genetic and/or epigenetic disease. Traditional epigenetic modifications, including the dysregulation of DNA methylation and histone modification, are causes of cancer, but chemical modifications of RNA were recently discovered to also cause cancer (Esteve-Puig et al., 2020). Breast cancer (BC) is the most common malignancy among women in the world (Siegel et al., 2020). Aberrant RNA modifications open a new era for studying the development and progression of BC (Chen et al., 2019).

Since the first modified nucleotides in RNAs were discovered in 1960 (Cohn, 1960), more than 170 distinct cellular RNA chemical modifications have been identified (Esteve-Puig et al., 2020), including pseudouridine (Ψ), N1-methyladenosine (m^1^A), 5-methylcytidine (m^5^C), N^6^-methyladenosine (m^6^A), 5-hydroxymethylcytosine (hm^5^C), etc. Among these, m^6^A is the most common and abundant posttranscriptional modification of messenger RNA (mRNA) and non-coding RNA (ncRNA). High-throughput sequencing revealed that one-third to one-half of mRNA transcripts had m^6^A modifications in human and mouse transcriptomes (Dominissini et al., 2012). Although m^6^A was first reported in 1974 (Desrosiers et al., 1974), little progress was made for decades (Reichel et al., 2019). Recently, the function of m^6^A has been gradually unveiled through advances in m^6^A detection techniques, such as liquid chromatography-tandem mass spectrometry (Fu et al., 2015), methylated RNA immunoprecipitation sequencing (Meyer et al., 2012), methylation individual nucleotide-resolution crosslinking immunoprecipitation (Linder et al., 2015), and single-molecule real-time sequencing (Zhu et al., 2016).

The m^6^A modification mainly identifies the conserved sequence RRACH (R = G/A, H = A/C/U). GGACU is one of the most common motifs (Harper et al., 1990). m^6^A modifications were previously believed to be mainly concentrated in the 3′-untranslated region (UTR), especially around the stop codon (Meyer et al., 2012). In recent years, researchers confirmed that the 5′-UTR (Meyer et al., 2015) and coding sequence (CDS; Mao et al., 2019) were also important for m^6^A modifications. Many studies have shown that they can regulate almost every stage of RNA metabolism, including alternative RNA splicing (Bartosovic et al., 2017; Louloupi et al., 2018), localization (Roundtree et al., 2017), translation efficiency (Wang et al., 2015b), mRNA stability (Wang et al., 2014), and protein expression (Yue et al., 2015).

N^6^-methyladenosine is involved in the regulation of many biological processes, such as the transition fate of mammalian embryonic stem cells (Batista et al., 2014) and circadian rhythms (Fustin et al., 2013; Zhong et al., 2018), and various diseases, such as obesity (Dina et al., 2007), infertility (Ding et al., 2018), type 2 diabetic mellitus (Shen et al., 2015), and many kinds of cancers (Chen et al., 2019). Compared with various studies that explored the interplay between m^6^A modifications and several types of cancers, studies of the role of m^6^A in BC are still in their infancy. Some conclusions of existing studies are controversial, thus underscoring the necessity to review and discuss the functions of m^6^A modifications and therapeutic strategies for BC.

This review focuses on the mechanism of m^6^A modifications, the roles of different m^6^A modulators in BC, especially their effect on proliferation, apoptosis, the cell cycle, and stemness, and therapeutic strategies for BC. This review will advance our understanding of the role of m^6^A modifications in the development and progression of BC.

## Mechanism of m^6^A Modification

There are mainly three kinds of mediator proteins that regulate m^6^A modifications: methyltransferase (writer), demethylase (eraser), and binding protein (reader). The regulation of m^6^A modifications is dynamically reversible, depending on the activity of the “writer” and “eraser.”

### m^6^A Methyltransferase (Writer)

**FIGURE 1:**
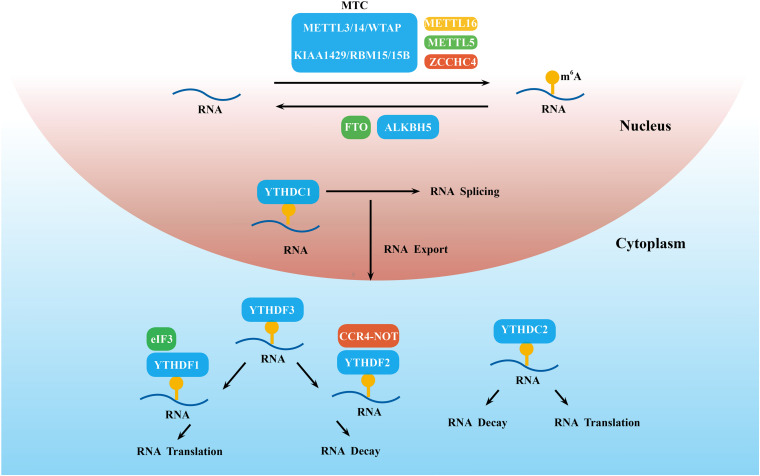
Detailed molecular mechanism of m^6^A modifications. These modifications are regulated by “writers” (the MTC, METTL5/16 and ZCCHC4), “erasers” (FTO and ALKBH5), and “readers” (YTHDC1-2 and YTHDF1-3), which install, remove, and recognize m^6^A and thereby regulate RNA splicing, export, decay, translation, and so on.

N^6^-methyladenosine modification is installed by the methyltransferase complex (MTC), which mainly consists of the methyltransferase like 3 (METTL3)/METTL14 heterodimer and Wilms’ tumor 1-associated protein (WTAP; Scholler et al., 2018; Figure 1). In this m^6^A MTC, METTL3 acts as the core catalytic subunit and transfers the methyl donor *S*-adenosylmethionine (SAM) to the adenine acceptor within the RRACH consensus motif. METTL14 colocalizes with METTL3 in nuclear speckles at a stoichiometric 1:1 ratio (Liu et al., 2014). METTL14 is responsible for stabilizing the structure of MTC, recognizing the substrate RNA sequence, and providing a binding platform (Wang P. et al., 2016; Wang X. et al., 2016). Huang et al. recently demonstrated that METTL14 can also recognize and bind H3K36me3. Thus, METTL14 facilitates m^6^A MTC binding to RNA polymerase II to mediate the m^6^A methylation of nascent RNA during transcription elongation (Huang H. et al., 2019). The combination of these histone and RNA modifications opens up new directions for epigenetics research. WTAP alone does not have any catalytic activity because it lacks the catalytic methylation domain, but its knockdown prominently decreases m^6^A levels (Liu et al., 2014). It regulates the level of m^6^A modification by interacting with the METTL3–METTL14 complex and promoting accumulation of the complex into nuclear speckles to efficiently methylate target RNAs (Ping et al., 2014). Other identified regulatory factors of MTC include KIAA1429/VIRMA (Yue et al., 2018), RBM15/15B (Patil et al., 2016; Knuckles et al., 2018), HAKAI (Ruzicka et al., 2017), and ZC3H13 (Knuckles et al., 2018).

Some newly discovered methyltransferase, such as METTL16, METTL5, and zinc finger CCHC-type containing 4 (ZCCHC4), can work alone and catalyze m^6^A on some structured RNAs, such as U6 snRNA (Aoyama et al., 2020), 18S rRNA (Leismann et al., 2020), and 28S rRNA (Ren et al., 2019), respectively (Figure 1).

### m^6^A Demethylase (Eraser)

N^6^-methyladenosine demethylases, including Fat mass and obesity-associated protein (FTO; Jia et al., 2012) and ALKB family protein 5 (ALKBH5; Zheng et al., 2013), can selectively remove the m^6^A modification and reverse the methylation process (Figure 1). The coordination between m^6^A methyltransferase and demethylase indicates that the m^6^A modification is dynamic and reversible. Although both FTO and ALKBH5 are members of the ALKB family, they have different substrates. Emerging evidence demonstrates that FTO is able to mediate the demethylation of m^6^A, m^6^Am, and m^1^A in the cell nucleus and cytoplasm (Wei et al., 2018). To date, m^6^A in mRNA was the main zymolytic substrate of FTO. Different from FTO, ALKBH5 is mainly localized in the nucleus and selectively removes the m^6^A methyl group (Mauer et al., 2017). This phenomenon reflects the complexity and specificity of the mechanism of m^6^A modifications. Additionally, ALKBH5 accelerated the process of both mRNA transfer from intranuclear to extranuclear and promoted translation initiation (Zheng et al., 2013), whereas METTL3 downregulation delayed this process (Fustin et al., 2013).

### m^6^A Binding Proteins (Reader)

Other types of m^6^A regulatory proteins, including YT521-B homology domain-containing family protein 1/2/3 (YTHDF1/2/3), YTH domain-containing proteins 1/2 (YTHDC1/2) (Haussmann et al., 2016), eukaryotic initiation factor 3 (elF3; Meyer and Jaffrey, 2017), the insulin-like growth factor-2 mRNA-binding protein (IGF2BP) family (Muller et al., 2019), and heterogeneous nuclear ribonucleoprotein (hnRNP) family (Zhao et al., 2017), recognize the m^6^A modification site and directly determine the fate of m^6^A-modified RNA (Casella et al., 2019; Figure 1).

YT521-B homology domain-containing family protein 1/2/3 and YTHDC1/2 share the same YTH domain and have a 50-times higher affinity for m^6^A mRNA than unmethylated mRNA (Theler et al., 2014). YTHDC1 was shown to be the major reader of nuclear m^6^A modifications and accelerated mature mRNA transportation from the nucleus to the cytoplasm by affecting mRNA splicing (Roundtree et al., 2017). To date, however, little is known about the function of YTHDC2 in m^6^A modifications. Hsu reported that YTHDC2 preferentially bound to m^6^A-marked RNA with the RRACH consensus motif and then increased translation efficiency by 52% but also reduced mRNA abundance (Hsu et al., 2017).

When mRNA arrives at the cytoplasm, m^6^A-methylated mRNA is mainly regulated by YTHDF1-3. YTHDF1 mainly binds to m^6^A sites near the stop codon in the 3′-UTR and then interacts with the translation initiation factor eIF3 to improve the translation efficiency of target mRNA (Wang et al., 2015b). In 2019, Lin et al. (2019) reported that YTHDF1, by binding with eEF-2 at m^6^A sites of the Snail CDS but not the 3′-UTR, promoted the translation of Snail. YTHDF2 was the first identified m^6^A-binding protein, which triggered mRNA degradation by recruiting the CCR4-NOT deadenylase complex (Du et al., 2016). The role of YTHDF3 is more complex. YTHDF3 acts in concert with YTHDF2 to accelerate mRNA decay (Shi et al., 2017), but it promoted the translation of m^6^A-modified RNA by cooperating with YTHDF1 (Li A. et al., 2017). IGF2BP family proteins (IGF2BP1, IGF2BP2, and IGF2BP3) are located in the cytoplasm and employ their K homology domains to identify the GG (m^6^A) C sequence. They promote the stability and storage of target mRNAs under both normal and stress conditions (Huang et al., 2018). Some other m^6^A readers, such as the HNRNP family and elF3, are under current exploration.

## m^6^A Modification in the Development and Progression of Breast Cancer

The majority of BC is sporadic and associated with alterations of genetics and epigenetics (Byler et al., 2014). Genetic alterations include mutations and copy number variations of certain genes. Conventional epigenetic remodeling consists of microRNA regulation, histone modification, and DNA methylation in BC (Rahman et al., 2019). m^6^A modifications open new directions for studying epigenetics. An increasing number of studies have revealed the importance of m^6^A in the development and progression of BC.

### The Role of m^6^A Methyltransferase in Breast Cancer

#### m^6^ A Methyltransferase Affects Breast Cancer Cells Through Several Molecular Mechanisms

**FIGURE 2:**
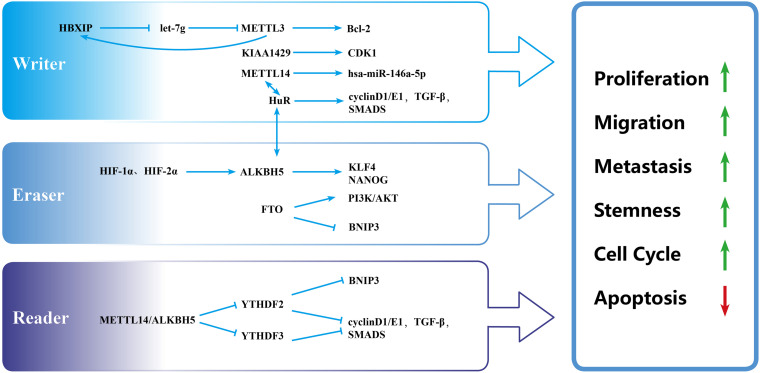
Biological function of m^6^A modulators in breast cancer. “Writers,” “erasers,” and “readers” promote cell proliferation, migration, metastasis, and stemness and inhibit apoptosis by regulating different targets.

In various cancer cells, the abnormal expression of METTL3 affects proliferation, invasion, metastasis, and the cell cycle (Zheng et al., 2019). In BC, METTL3 mainly acts as an oncogene (Figure 2). For example, METTL3 overexpression in transformed cells enhanced proliferation and migration ability, suggesting that the downregulation of m^6^A modification acts as a brake during malignant transformation (Fry et al., 2018). B-cell lymphoma 2 (Bcl-2) is an anti-apoptotic protein (Konig et al., 2019) that has been shown to be a potential target of METTL3 in BC tissues and cells. The knockdown of METTL3 reduced the expression of Bcl-2, repressed the proliferation of MDA-MB-231 and MCF-7 cells, accelerated their apoptosis, and inhibited the growth of transplanted tumors *in vivo* (Wang et al., 2020).

A few studies have shown that hepatitis B x-interacting protein (HBXIP) is highly expressed in BC as an oncogene (Yue et al., 2013; Liu et al., 2014). METTL3 was positively linked with HBXIP in BC tissues and cells. In BC cells, HBXIP inhibited the expression of let-7g, which repressed the expression of METTL3 by targeting the 3′-UTR of METTL3 (Cai et al., 2018). Additionally, METTL3 upregulated the expression of HBXIP by stimulating the m^6^A modification of HBXIP and thus formed a positive feedback regulatory loop of HBXIP/let-7g/METTL3/HBXIP, leading to the proliferation of BC cells (Cai et al., 2018). Cui et al. (2017) reported that the knockout of METTL3 and METTL14 promoted the growth, self-renewal, and tumorigenesis of glioblastoma stem cells (GCS) partly by upregulating BRCA2. BRCA2 was tightly related to hereditary BC (Paul and Paul, 2014). However, whether METTL3 and METTL14 regulate the development and progression of BC through BRCA2 is unknown. Altogether, Bcl-2, HBXIP, and BRCA2 are all potential targets of METTL3. We speculate that other targets of METTL3 will be uncovered as regulators of BC.

METTL14 is also closely related to the progression of BC (Figure 2). The knockdown of METTL14 reduced the long-term viability, migration, and invasion of BC cell lines (MDA-MB-231, MDA-MB-468, and BT549) and inhibited tumor growth in tumor xenograft models (Panneerdoss et al., 2018). BC cells were arrested in the G1–S phase when METTL14 was silenced. Mechanistically, METTL14 and the RNA-binding protein HuR form a potential positive feedback loop that regulates transforming growth factor-β (TGF-β) signaling pathway genes and cell cycle-associated genes (cyclin D1 and cyclin E1) and exhibit hyper m^6^A with lower expression compared with scrambled control cells (Panneerdoss et al., 2018). m^6^ A also affects BC cells through regulating the noncoding RNAs expression. Yi et al. found that METTL14 overexpression promoted the migration and invasion of MDA-MB-231 and MCF-7 cells through the upregulation of hsa-mir-146a-5p (Yi et al., 2020). However, Wu et al. reported that the overexpression of METTL14 in MDA-MB-231 cells inhibited cell viability, clone formation, and cell migration (Wu et al., 2019). METTL14 appears to play opposing roles in the same cell line. Whether other factors, such as the tumor microenvironment, impact METTL14 function deserves further exploration.

The role of methyltransferase KIAA1429 in BC had been preliminarily elucidated. A previous study (Qian et al., 2019) showed that KIAA1429 promoted BC cell proliferation and the epithelial–mesenchymal transition (EMT) in MCF-7 and SUM1315 cell lines. This may be caused by an increase in stability of the cell cycle regulator cyclin-dependent kinase 1 (CDK1) mRNA in an m^6^A-independent manner (Qian et al., 2019). The roles of other methyltransferases in BC have rarely been reported and deserve further study.

#### m^6^A Methyltransferase Exhibits Abnormal Expression in Breast Cancer Tissues and Is Related to Certain BC Subtypes

Breast cancer tissues exhibit the dysregulation of m^6^A methyltransferase compared with normal breast tissues, although m^6^A methyltransferase levels vary (Table 1). The mRNA expression of classic methyltransferases (i.e., METTL3, METTL14, and WTAP) was reported to be either upregulated (Cai et al., 2018; Wang et al., 2020; Yi et al., 2020) or downregulated (Liu et al., 2019; Wu et al., 2019). Moreover, their mRNA levels were sometimes inconsistent with protein levels. Liu performed immunohistochemical staining in 20 matched BC and adjacent normal tissues with a BC tissue microarray (TMA) and found the upregulation of WTAP (*p* = 0.002), KIAA1429 (*p* < 0.001), and RBM15 (*p* = 0.012) but no significant changes in METTL3, METTL14, METTL16, or RBM15B in BC specimens (Liu et al., 2019), which was different from mRNA expression.

**TABLE 1:** Different expression levels and potential prognostic value of m^6^A modulators in breast cancer (BC).

**Genes**	**Up-/Down-regulation of expression**	**Methods of Detection**	**Correlation with prognosis**	**References**
METTL3	Upregulated	GEPIA*/qRT-PCR/Western Blot	Unfavorable survival rate	Cai et al., 2018; Wang et al., 2020
	Downregulated	Oncomine*/TCGA*/qRT-PCR	Good RFS*	Wu et al., 2019
METTL14	Upregulated	qRT-PCR	Good RFS	Yi et al., 2020
	Downregulated	Oncomine/TCGA/		Liu et al., 2019; Wu et al., 2019
WTAP	Upregulated	IHC		Liu et al., 2019
	Downregulated	Oncomine/TCGA/		Wu et al., 2019; Liu et al., 2019
RBM15	Upregulated	TCGA/IHC		Liu et al., 2019
RBM15B	No significance	TCGA/IHC	Good OS*/good RFS	Liu et al., 2019
KIAA1429	Upregulated	TCGA/IHC	Poor OS	Liu et al., 2019
METTL16	Downregulated	TCGA	Poor OS	Liu et al., 2019
FTO	Upregulated	IHC	Poor OS, associated with the risk of BC	Tan et al., 2015; Chen et al., 2018; Gholamalizadeh et al., 2020
	Downregulated	Oncomine/TCGA/HPA*/qRT-PCR	Poor RFS	Liu et al., 2019; Wu et al., 2019
ALKBH5	Upregulated	TCGA/HPA/qRT-PCR/IHC		Liu et al., 2019; Wu et al., 2019
	Downregulated	Oncomine/TCGA	Poor RFS	Liu et al., 2019; Wu et al., 2019
YTHDF1	Upregulated	TCGA/IHC	Poor OS	Liu et al., 2019
YTHDF2	Upregulated	TCGA/IHC	oncogene	Chen et al., 2018; Liu et al., 2019
YTHDF3	Upregulated	IHC	Poor OS/independent predictor	Liu et al., 2019
YTHDC1	Downregulated	TCGA/HPA		Liu et al., 2019
HNRNPC	Upregulated	TCGA/IHC/HPA		Liu et al., 2019
HNRNPA2B1	Upregulated	TCGA/IHC		Liu et al., 2019

Note: GEPIA*, Oncomine*, TCGA*, and HPA* are online databases. RFS*, relapse-free survival; OS*, overall survival.

The expression of m^6^A methyltransferase also correlated with molecular subtypes of BC (Table 2). METTL3 and METTL14 were highly expressed in normal breast-like and luminal A/B BC, but WTAP was mainly expressed in basal-like BC, based on results from the Oncomine and The Cancer Genome Atlas (TCGA) database (Wu et al., 2019). Inflammatory BC (IBC) is a rare and aggressive form of BC. The triple-negative subtype of IBC (TN-IBC) is substantially more metastatic and fatal than TN-non-IBC. In an analysis of differentially regulated genes in TN-IBC and TN-non-IBC, ZC3H13 (i.e., a member of the MTC) was among the top 10 genes and downregulated in TN-IBC (Funakoshi et al., 2019). These data are summarized in Table 2.

**TABLE 2:** Correlation between m^6^A modulators and molecular typing of BC.

**Gene (regulated expression)**	**Molecular subtypes**	**References**
METTL3 (up)	Normal breast-like and luminal A/B	Wu et al., 2019
METTL14 (up)	Normal breast-like and luminal A/B	Wu et al., 2019
WTAP (up)	Basal-like	Wu et al., 2019
ZC3H13 (down)	TN-IBC*	Funakoshi et al., 2019
FTO (up)	ER (+)/PR (+) patients; Her2+; HR-/HER2+	Tan et al., 2015; Chen et al., 2018; Wu et al., 2019
FTO (down)	HER2 (+)	Wu et al., 2019
ALKBH5 (up)	ER (+) or PR (+) patients	Wu et al., 2019

*TN-IBC, triple-negative inflammatory breast cancer.*

#### The Atypical Expression of m^6^A Methyltransferase Is Closely Related to BC Prognosis

Studies have reported conflicting reports on the role of m^6^A methyltransferase in the prognosis of BC (Table 1). Based on data from the TCGA-BC cohort, the higher expression of RBM15B (*p* = 0.014, 95% confidence interval: 0.48–0.94) significantly correlated with favorable overall survival (OS), whereas the high expression of KIAA1429 (*p* = 0.032, 95% confidence interval: 1.03–1.96) and METTL16 (*p* = 0.02, 95% confidence interval: 1.06–2.02) correlated with poor OS. With regard to relapse-free survival (RFS), the overexpression of RBM15B (*p* = 0.021, 95% confidence interval: 0.54–0.96) correlated with good RFS. However, other “writers” did not critically affect the survival rate (Liu et al., 2019). Another study, based on the bc-GenExMiner 4.0 database, found that the high expression of METTL3, METTL14, and WTAP correlated with good metastasis relapse (MR)-free survival in all BC patients (Wu et al., 2019). However, a separate study that conducted a Kaplan–Meier test revealed that BC patients with high METTL3 expression had unfavorable survival rates (Wang et al., 2020).

### Role of m^6^A Demethylase in Breast Cancer

#### m^6^A Demethylases Affects Breast Cancer Cells via Different Molecules and Signaling Pathways

At the cellular level, FTO promoted the proliferation and mammosphere formation and suppressed cell apoptosis by inhibiting BCL2/adenovirus E1B 19-kDa protein-interacting protein 3 (BNIP3; i.e., a pro-apoptosis protein of the Bcl-2 family) in BC (Niu et al., 2019) (Figure 2). FTO demethylated the 3′-UTR of BNIP3 mRNA and promoted the degradation of BNIP3 mRNA in a YTHDF2-dependent manner. In MDA-MB-231 and MCF-7 cell lines, silencing BNIP3 alleviated the inhibition of cell proliferation that was mediated by FTO knocking down. In 4T1 cells, BNIP3 knockdown significantly weakened FTO-accelerated tumor growth and metastasis in a subcutaneous implantation model and tumor metastasis model in Balb/c mice. Additionally, FTO upregulated glycolysis and energy metabolism through the PI3K/AKT pathway in MDA-MB-231 and MCF-7 cells (Liu et al., 2017). The PI3K/AKT signaling pathway has a close relationship with proliferation, metabolism, immune response regulation, motility, and survival (Ortega et al., 2020). Therefore, FTO may exert important actions through the PI3K/AKT pathway, which deserves further study.

ALKB family protein 5 can promote the growth and metastasis of BC cells (Figure 2). The silencing of ALKBH5 inhibited the viability, migration, and invasion of BC cell lines and tumor growth in a tumor xenograft model in mice (Panneerdoss et al., 2018), similar to METTL14 knockdown that was mentioned above. In MDA-MB-231 cells, the knockdown of ALKBH5 promoted the m^6^A modification of mRNA and inhibited the ability of survival, clone formation, and cell migration (Wu et al., 2019). In a mouse model of the orthotopic transplantation of BC tumors, ALKBH5-deficient MDA-MB-231 cells developed fewer tumors compared with the control group (43% vs. 100%), and only a few small lung metastases were found in the ALKBH5 knockdown group, thus confirming that ALKBH5 promoted the initiation of BC and lung metastasis (Zhang et al., 2016b). Under hypoxic conditions, hypoxia-inducible factor 1α (HIF-1α) and HIF-2α induced overexpression of the m^6^A demethylase ALKBH5, which increased demethylation of the 3′-UTR of pluripotent factor NANOG mRNA and enhanced its protein stability (Zhang et al., 2016a). Therefore, ALKBH5 mediates BC stem cell (BCSC) transformation by NANOG in a HIF-dependent manner. Moreover, Zhang et al. (2016b) showed that hypoxia induced expression of the important oncogene ZNF217, which restrained m^6^A RNA methylation by blocking METTL3. The knockdown of ZNF217 and ALKBH5 increased m^6^A modification and inhibited hypoxia-induced expression of the pluripotent stem cell factors NANOG and KLF4, thereby suppressing the pluripotency of BC cells (Zhang et al., 2016b). Based on these studies, we speculate that silencing ALKBH5 may be an effective therapeutic strategy that can inhibit proliferation, metastasis, and stemness in BC.

#### m^6^A Demethylase Exhibits Abnormal Expression in Breast Cancer Tissues and Is Related to Certain BC Subtypes

To explore the clinical significance of FTO and ALKBH5 demethylases in BC, various studies have detected their expression levels based on three independent databases [Oncomine, TCGA, and the Human Protein Atlas (HPA)] in clinical BC specimens (Table 1). All of these databases indicated that FTO mRNA is significantly reduced in BC (Liu et al., 2019; Wu et al., 2019). ALKBH5 mRNA was either increased (Liu et al., 2019; Wu et al., 2019) or decreased (Wu et al., 2019) compared with normal tissue. At the protein level, immunohistochemical staining showed that ALKBH5 and FTO expression was high in BC (Tan et al., 2015; Liu et al., 2019; Niu et al., 2019). Notwithstanding these studies, no definitive conclusions can be drawn about the expression level of m^6^A demethylase in BC. We briefly summarize these data in Table 1.

A correlation was found between the expression level of demethylase and molecular subtypes of BC (Table 2). ALKBH5 and FTO mRNA expression significantly increased in estrogen receptor (ER)-positive or progesterone receptor (PR)-positive patients, whereas FTO mRNA expression decreased in human epidermal growth factor receptor 2 (HER2)-positive patients, based on clinicopathological parameters from the bc-GenExMiner 4.0 database (Wu et al., 2019). Tan reported that FTO was highly expressed in hormone receptor (HR)-negative and HER2-positive BC patients, based on the immunohistochemical staining of specimens from 79 infiltrating ductal breast cancer (IDBC) patients (Tan et al., 2015). In another study that analyzed data from Gene Expression Omnibus (GEO) database (GSE9014), FTO expression was upregulated in HER2-positive BC (Niu et al., 2019).

#### The Atypical Expression of m^6^A Demethylase Is Related to BC Prognosis

Findings of the prognostic value of FTO and ALKBH5 in BC have been variable (Table 1). Kaplan–Meier analysis, meta-analysis, and univariate Cox analysis indicated that decreases in FTO mRNA levels suggest poor RFS, based on data from the bc-GenExMiner 4.0 database, whereas ALKBH5 was not significantly related to prognosis (Wu et al., 2019). Another study found that high FTO expression was directly related to poor OS in ER-negative BC patients and advanced BC patients (Niu et al., 2019), based on the Genomics Analysis and Visualization Platform.^1^ FTO gene polymorphisms, such as rs9939609 and rs1477196, were associated with the risk of BC (Gholamalizadeh et al., 2020). This association may be affected by ER/PR status, tumor stage, and body mass index (BMI) (Gholamalizadeh et al., 2020).

### The Role of m^6^A-Binding Protein in Breast Cancer

Compared with other m^6^A modulators, less research on the effect of m^6^A-binding protein in BC has been conducted (Tables 1, 2). Liu et al. (2019) reported that YTHDF1 (*p* < 0.001), YTHDF2 (*p* = 0.022), HNRNPC (*p* < 0.001), and HNRNPA2B1 (*p* < 0.001) were upregulated, and YTHDC1 (*p* = 0.013) was downregulated in BC samples, based on RNA-seq data from TCGA. At the protein level, immunohistochemical staining from 20 pairs of BC and adjacent matched normal breast tissue samples showed that YTHDF1-3, HNRNPC, and HNRNPA2B1 were significantly overexpressed in neoplastic tissues. No significant difference in YTHDC1 was found (Liu et al., 2019). However, HPA (without YTHDF1 or YTHDF3 data) analysis showed that HNRNPC and YTHDC1 expression was high in BC, whereas YTHDF2 and HNRNPA2B1 expression was not significantly different between normal and BC tissues (Liu et al., 2019). Further investigations of the prognostic value of m^6^A-binding protein in BC revealed that the high expression of YTHDF1 (*p* = 0.049, 95% confidence interval: 1–1.91) and YTHDF3 (*p* < 0.001, 95% confidence interval: 1.28–2.49) was related to poor OS, based on clinical data from TCGA. Furthermore, YTHDF3 overexpression was deemed to be an independent predictor of poor OS in BC patients, based on univariate and multivariate analyses (Liu et al., 2019). Studies of the mechanism of regulation found that YTHDF2 participated in the degradation of BNIP3 by binding to potential m^6^A sites in the 3′-UTR in BC (Figure 2). BNIP3 acts as a tumor suppressor, and YTHDF2 may play an oncogenic role by regulating BNIP3 (Niu et al., 2019).

Furthermore, significant crosstalk among YTHDF3, METTL14, and ALKBH5 has been reported (Panneerdoss et al., 2018). YTHDF3 levels significantly increased in BC cells with METTL14 and ALKBH5 knockdown, detected by RNA-seq and Western blot. It was further confirmed that such crosstalk regulated cancer cell growth and progression in rescue experiments in which YTHDF3 was knocked down in MRTTL14- and ALKBH5-depleted BC cells. We speculate that additional crosstalk exists among other m^6^A molecules, which requires further study. Overall, m^6^A modifications are involved in the development and progression of BC, the relevant mechanisms of which are summarized in Figure 2.

## m^6^A-Targeting Drugs Suppress the Progression of Breast Cancer

The dysregulation of m^6^A modifications is linked to various diseases, especially cancer. The development of small-molecule m^6^A-targeting drugs is an attractive therapeutic strategy. Some m^6^A inhibitors, such as cycloleucine (Wang et al., 2015a) and 3-deazaadenosine (Chen et al., 2018; Yi et al., 2020), were shown to nonspecifically reduce m^6^A levels by dose-dependently inhibiting SAM activity. During tumor progression, m^6^A modification acts as a “dual-edged sword” with the controversial role of some m^6^A modulators. The discovery of specific m^6^A regulators may contribute to more effective treatment strategies for cancer.

Among the m^6^A regulators, FTO was the first m^6^A demethylase that was discovered in 2011 (Jia et al., 2011), which has attracted much interest because of its involvement in obesity and obesity-induced metabolic diseases (Zhao et al., 2014) and the occurrence, development, and prognosis of many kinds of cancer, such as melanoma (Yang et al., 2019), acute myeloid leukemia (AML; Li Z. et al., 2017), glioblastoma (Cui et al., 2017), lung carcinoma (Li et al., 2019a), hepatocellular carcinoma (Li et al., 2019b), and BC (Niu et al., 2019). FTO has been well studied, and several inhibitors of FTO have been developed with regard to their actions against m^6^A modifications (Deng et al., 2018). To date, such FTO inhibitors include entacapone (which lowers fasting blood glucose levels and reduces body weight in diet-induced obese mice) (Peng et al., 2019), R-2HG (which inhibits proliferation/viability and promotes cell cycle arrest and apoptosis in FTO-high cancer cells) (Su et al., 2018), rhein (which suppresses axon elongation in axons) (Yu et al., 2018), meclofenamic acid (MA; which inhibits the proliferation of BC cells) (Kovala-Demertzi et al., 2009), MA2 (the ethyl ester form of MA; which arrests the G1/S transition and inhibits cell proliferation in germ cells) (Huang T. et al., 2019), FB23 and FB23-2 (which suppress proliferation and promote the differentiation and apoptosis of AML cells *in vitro* and *in vivo*) (Huang Y. et al., 2019), and CS1 and CS2 (which attenuate the self-renewal ability and reprograming immune response of leukemia cells) (Huang et al., 2015). These inhibitors have been shown to have different mechanisms of action, and the effects of rhein and MA have been explored in BC.

Rhein was the first discovered natural small-molecule inhibitor of FTO. It competitively binds the catalytic region of FTO and upregulates cellular m^6^A levels *in vitro* (Chen et al., 2012). In axons, FTO inhibition by rhein or FTO knockdown by siRNA downregulated the local translation of GAP-43 mRNA and resulted in the suppression of axon elongation (Yu et al., 2018). Before rhein was found to be an inhibitor of FTO, it was shown to have antitumor effects. Rhein inhibited MCF-7 and MDA-MB-435 BC cell viability and growth by suppressing vascular endothelial growth factor (VEGF)- and endothelial growth factor (EGF)-induced activation of the PTEN/PI3K/AKT/mTOR and MAPK/ERK pathways in BC (Fernand et al., 2011). Rhein also had anti-proliferative and pro-apoptosis ability in MCF-7 cells and HER2-overexpressing MCF-7 cells (MCF-7/HER2). The rhein-induced inhibition of cell growth was associated with caspase-9-mediated apoptosis, and reactive oxygen species-mediated activation of the NF-κB and p53 signaling pathways also participated in this process (Chang et al., 2012). In 2019, one study evaluated rhein’s function *in vivo*. Rhein enhanced the apoptotic effect of atezolizumab on 4T1 BC cells (Shen et al., 2019). Treatment with a combination of rhein (10 mg/kg) and atezolizumab (10 mg/kg) inhibited 4T1 xenograft tumor growth and increased serum levels of tumor necrosis factor α and interleukin-6, the CD8^+^ T-cell ratio, apoptotic factors (e.g., caspase-3, caspase-8, and caspase-9), and Bax/Bcl-2 mRNA levels compared with rhein or atezolizumab treatment alone. However, these experiments did not clarify the role of m^6^A modifications in the inhibitory effect of rhein in BC, which deserves further exploration.

Meclofenamic acid is a nonsteroidal anti-inflammatory drug that was originally approved by the United States Food and Drug Administration. It competes for FTO binding sites on single-stranded DNA (ssDNA) and directly interacts with FTO protein in HeLa cells to inhibit the catalytic activity of FTO and improve levels of m^6^A modifications in RNA (Huang et al., 2015). Meclofenamic acid inhibited the proliferation of MCF-7 BC cells, with low toxicity (Kovala-Demertzi et al., 2009). However, whether this result can also be achieved by changing the level of m^6^A modification remains unknown and requires further investigation.

Although numerous small-molecule m^6^A regulators have been identified, further studies are needed to define their specificity and adverse effects for the treatment of BC *in vitro* and *in vivo*. Furthermore, multiple combinations of existing medical approaches with m^6^A-targeting drugs may be viable options for the treatment of BC.

## Conclusion

m^6^A modifications are one of the most common modifications of mRNA and, most importantly, dynamically reversible. Several studies have shown that m^6^A modifications are closely related to many human diseases, including cancer. However, the relationship between m^6^A modifications and BC has not been fully elucidated. m^6^A modifications play a complex role in expression levels, genotyping, prognosis, cell proliferation, and metastasis in BC. The controversial findings on the role of some m^6^A modulators in BC may be attributable to the different test platforms that were employed or inconsistent proteomics and transcriptomics analyses or selective bias of clinical specimens. Most studies of m^6^A modifications in BC have been performed *in vitro*. Therefore, more *in vivo* evidence is required to reveal the regulatory mechanism of m^6^A modifications in BC.

Based on the significant role of m^6^A modifications in oncogenesis and progression that has been reported, the development of novel m^6^A regulators may be the new therapeutic options for malignant tumors. FTO, which was originally found to be involved in obesity and fat metabolism, was the first discovered m^6^A demethylase. The antitumor effects of some agents that target FTO have been reported in BC. However, little is known about their exact mechanism of action and side effects. Whether these inhibitors influence other RNA modifications, such as m^1^A, m^6^Am, m^5^C, and hm^5^C, is unknown. More studies are also needed to demonstrate their effectiveness *in vivo*. Combination therapies, such as combining chemotherapy, radiotherapy, and immune checkpoint blockade with proper m^6^A inhibitors, may hold promise for the treatment of BC, particularly those that have failed in routine treatment. Based on the oncogenic role of FTO in several cancers, inhibitors that target FTO have attracted scientific interests, but more information about its role in BC is needed. YTHDF3, an independent prognostic factor in BC, may be another target that merits further investigation.

N^6^-methyladenosine modifications in the progression of BC are still being discovered. We expect that substantial progress will be made in further revealing the role of m^6^A modifications in BC. Further advances in technology will likely contribute to the development of new, promising, and unexpected therapeutic strategies for the treatment of BC in coming years.

## Author Contributions

MW and J-WB designed the project and wrote the manuscript. Y-QZ, LN, and H-YC performed the literature searches in PubMed, Medline, and Google Scholar. G-JZ arranged and supervised the overall project. All authors contributed to the article and approved the submitted version.

## Conflict of Interest

The authors declare that the research was conducted in the absence of any commercial or financial relationships that could be construed as a potential conflict of interest.

## Footnotes

1 https://hgserver1.amc.nl
